# 0.46 Terahertz wave irradiation inhibit transcription reaction in liposomes

**DOI:** 10.1038/s41598-025-03869-w

**Published:** 2025-05-28

**Authors:** Gakushi Tsuji, Yuusuke Yamaguchi, Masaya Oki

**Affiliations:** 1 Department of Applied Chemistry and Biotechnology, Graduate School of Engineering, University of Fukui, 3-9-1 Bunkyo, Fukui-shi, Fukui, 910-8507 Japan; 2 Life Science Innovation Center, University of Fukui, 3-9-1 Bunkyo, Fukui-shi, Fukui, 910-8507 Japan; 3 Research Center for Development of Far-Infrared Region, University of Fukui, 3-9-1 Bunkyo, Fukui-shi, 910-8507 Fukui, Japan

**Keywords:** Liposome, Transcription, Flow cytometry, Gyrotron, Terahertz-wave, Membranes, Synthetic biology, Biophysical chemistry

## Abstract

Terahertz waves are absorbed by hydrogen bonds between water molecules and proteins, and low-frequency terahertz waves, in particular, have been reported to cause changes in protein function and inhibit cell division. In this study, we established an experimental system to irradiate liposomes containing T7 RNA polymerase for in vitro transcription reactions with terahertz waves in the absence of external fluid, and analyzed the resulting changes in transcription reaction efficiency. Terahertz wave irradiation at 460 GHz did not alter the shape of the liposomes, but the intraliposomal transcription reaction was non-thermally inhibited during irradiation, regardless of the energy per pulse. After irradiation, the transcription reaction efficiency was found to be higher than in non-irradiated samples. Since our experimental system allows for the analysis of a wide range of terahertz wave frequencies, pulse widths, pulse intervals, and energy levels, we can comprehensively explore the effects of terahertz waves on living organisms, an area that has been challenging to study in the past. This capability significantly broadens the potential applications of terahertz waves in future research.

**Supplementary Information:**

The online version contains supplementary material available at 10.1038/s41598-025-03869-w.

## Introduction

Biochemical reactions in cells occur in an aqueous environment with high concentrations of proteins, with various enzymatic processes, including transcription and translation, are actively functioning. In recent years, terahertz waves above 100 GHz have shown promise in mobile communication systems and various scientific applications. Terahertz wave sources capable of emitting across a wide range of frequencies, power levels, and pulse widths are currently under development. Terahertz waves have been shown to influence hydrogen bonds between water molecules and biological macromolecules^1^, thereby affecting intracellular functions. For example, irradiation of a human skin tissue model with terahertz waves at 0.1–2.0 THz has been shown to cause DNA double-strand break (DSB)^2^. This DSB effect also depends on the cell type. For instance, 2.3 THz terahertz waves have been irradiated on human stem cells, E. coli, and Salmonella typhimurium, with no DNA damage response observed following irradiation^3,4^. Additionally, irradiation of purified actin protein with 0.46 THz (460 GHz) terahertz waves promotes actin polymerization, which in turn inhibits the division of human cultured cells in vivo^5^. It has also been reported that irradiation of the K12 JM109 strain of E. coli with terahertz waves (1 ~2 THz) activates the expression of genes that regulate cell division in subsequent cultures, leading to morphological abnormalities in the cells^6^.

Although terahertz waves are known to affect cells by causing DNA damage and inhibition cell division, many technical challenges remain in elucidating the detailed effects of terahertz wave irradiation on cells. One reason for this is that temperature can significantly influence organisms and their metabolism. It has been indicated that a temperature difference of no more than 0.1 °C between a non-irradiated sample and an irradiated sample is required to detect the non-thermal effects of terahertz waves^7^. To address these technical difficulties, in vitro reconstructions are an effective experimental system, as they allow for the analysis of biochemical reactions without the confounding physiological effects. However, detecting effects in cells within an in vitro reconstituted system can be challenging because terahertz waves are absorbed by hydrogen bonds between water molecules.

In this study, liposomes (giant unilamellar vesicles, GUVs) were used as cell-like compartments. GUVs can encapsulate various cellular functions, including the reconstitution of biochemical reactions such as RNA replication^8^, transcription^9,10^ translation^11–14^, RNA self-replication^15^, and the elongation of tubulin filaments^16^ and actin filaments^17,18^. We developed a method for irradiating THz waves using a gyrotron^19^ on GUVs encapsulating in vitro transcription reactions. Interestingly, the transcription reaction in liposomes was suppressed during the irradiation with THz waves, but it was accelerated afterward. These results suggest that terahertz wave irradiation not only causes DNA damage but also alters the hydration state of the enzyme’s active site^20^, inhibiting the polymerization reaction of nucleic acids during transcription.

## Results

### Development of terahertz wave irradiation system for liposomes

**Fig. 1:**
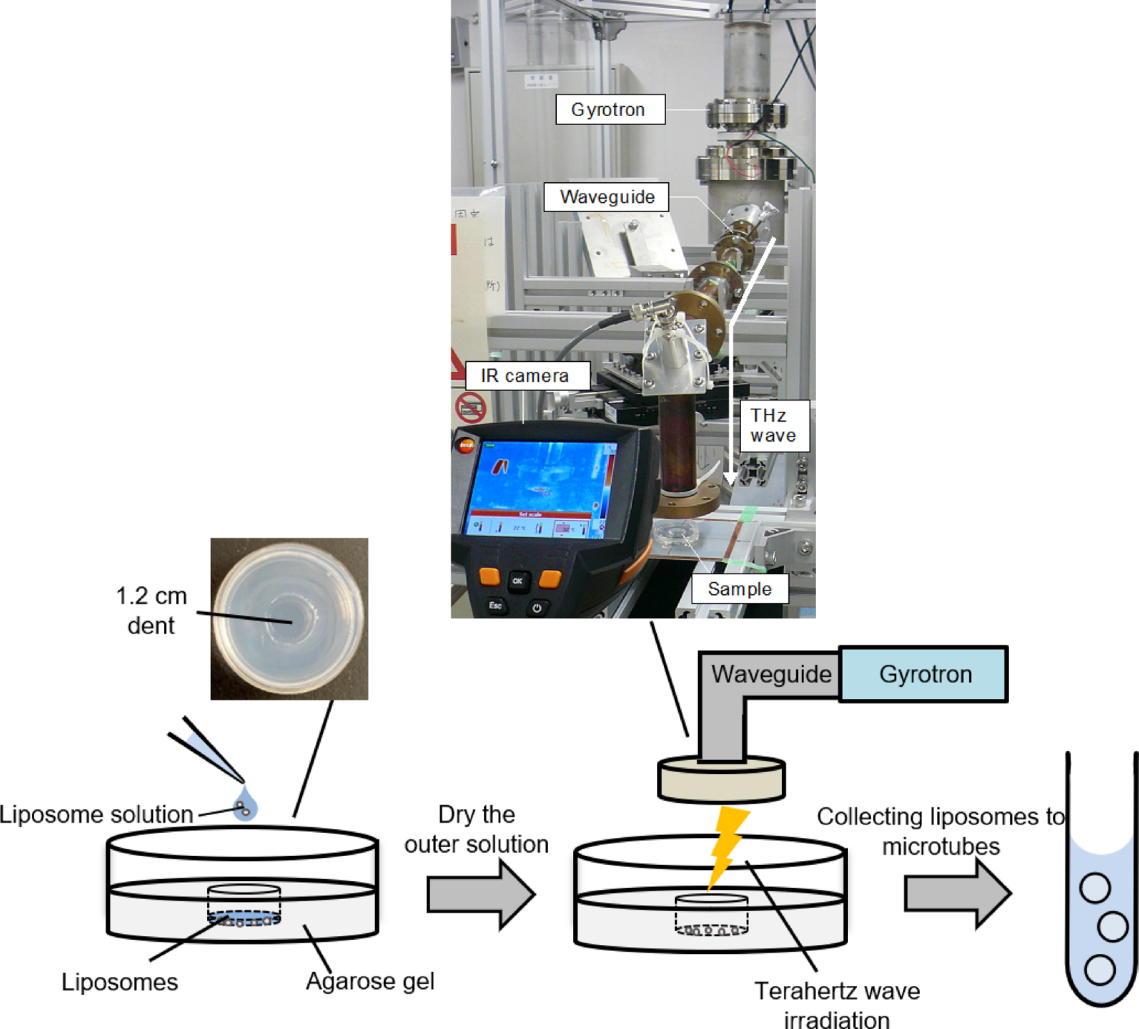
Schematic illustration of the experimental condition The liposome solution is applied to the dent of the agarose gel, and the terahertz wave is irradiated after the outer solution is dried. The liposomes are collected in a microtube by adding the outer solution to the dent in the gel.

**Fig. 2:**
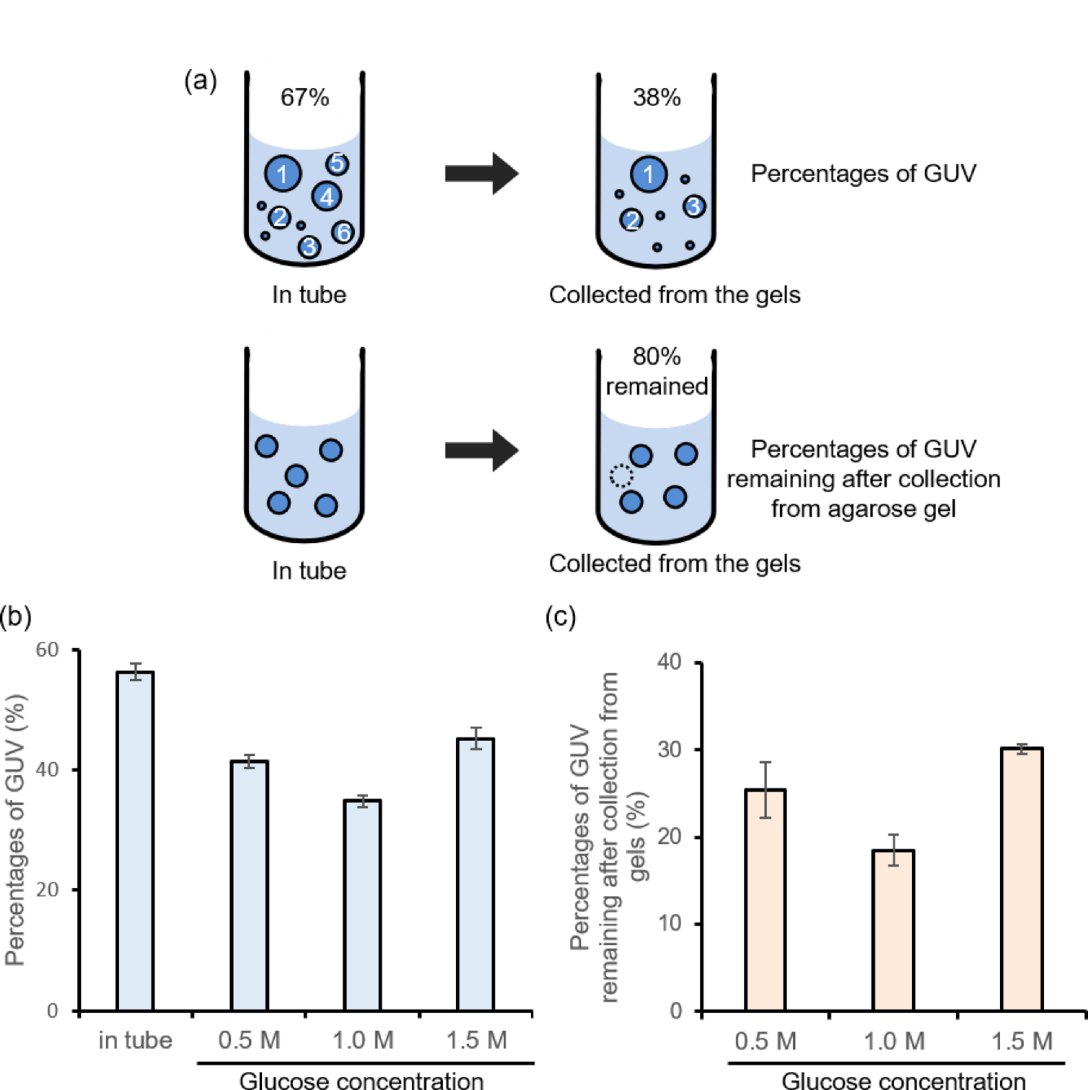
Analysis of the collected liposomes from agarose gel. **a** Schematic representation of the analysis of percentages of GUV which were shown in (**b**), and percentages of GUV remained after F/T which were shown in (**c**). **b** The average ratios of GUVs before and after applied on the agarose gel. The error bars indicate the standard errors (*n* = 3). **c** The average numbers of GUVs in a microliter of liposome solution before and after applied on the agarose gel. The error bars indicate the standard errors (*N* = 3).

First, we developed a method for irradiating terahertz waves on liposomes without an outer solution. We used a water-in-oil (w/o) emulsion transfer method^8,21^ to prepare giant unilamellar vesicles (GUVs) as model compartments. Since terahertz waves are predominantly absorbed by water, irradiating biochemical reactions in liposomes dispersed in an outer solution is challenging. It has been reported that terahertz waves irradiated through plastic plates to adherent cultured cells successfully demonstrated a non-thermal inhibitory effect on cell division^5^. Since liposomes are unstable in a dried condition^22^, we developed an irradiation system for liposomes without an outer solution by using agarose gels (Fig. 1). The agarose gel contained the components of the outer solution to prevent the liposomes from bursting. A dent is required to control liposome localization and facilitate efficient collection after terahertz irradiation when adding the outer solution. The distance from the waveguide exit to the irradiated area was approximately 1.5 cm. In this system, we placed the liposome solution in the dent of the gel, allowed it to dry, and then irradiated it with terahertz waves (Fig. 1). The IR camera was used to measure the temperature during irradiation. After irradiating with terahertz waves, the outer solution was added, and the liposomes were collected into microtubes. To optimize osmotic pressure for efficient collection of liposomes, liposome solutions were added to agarose gels with varying concentrations of glucose, and the liposomes were then collected from the gels. Percentages of GUVs before and after exposure to the gels, as well as the ratio of the number of GUVs collected from the gels compared to those before adding them to the gels, were determined by flow cytometry (FCM) analysis Fig. 2a). Although the percentage of GUVs decreased after being added to the gels (Fig. 2b, Fig S1) with 70% of GUVs breaking (Fig. 2c), a sufficient number of liposomes were still collected using this method. Based on these results, we selected a 1.5 M glucose concentration for use with the irradiation system, as it yielded the highest percentages of both GUVs and the number of remaining GUVs.

### Effects of terahertz wave irradiation on liposome size and internal contents

Studies on biochemical reactions encapsulated in liposomes composed of POPC^23^ have shown stability at temperatures ranging from room temperature to 37°C^9,15^. However, no studies have yet irradiated cell-sized GUVs with terahertz waves and performed quantitative analyses of liposome size, GUV ratios, and internal content leakage before and after terahertz irradiation. First, we established three terahertz wave irradiation conditions, as detailed in Table 1, with adjustments to peak power, pulse duration, and pulse repetition rate (Fig. S2) to ensure equal irradiation energy at the two frequencies. In our experimental system, the temperature of the agarose gel exposed to terahertz wave irradiation was measured using an IR camera and found to be 27.5–28 °C. To determine the optimal irradiation time for the terahertz waves and assess whether RNA transcription occurred in liposomes under these temperature conditions, we prepared liposomes encapsulating an RNA transcription reaction and added them to the agarose gel. After incubation at 28 °C for 1 or 3 h, the liposomes were collected from the gel, and the amount of RNA was quantified using flow cytometry (FCM) with SYBR Green II fluorescence. The results showed that after 1 h, the fluorescence signal was comparable to that of the non-reactive condition maintained on ice, while after 3 h, the fluorescence intensity had increased (Fig. S3).　According to these results, two irradiation conditions need to be considered: 1 h irradiation, where no RNA transcription occurs during irradiation and the transcription efficiency after irradiation can be compared, and 3 h irradiation, where some transcription is expected during irradiation, enabling reaction efficiency both during and after irradiation. Therefore, we examined morphological changes in liposomes by conducting experiments under both 1 h and 3 h irradiation conditions. Real-time temperature measurements using the IR camera showed that the temperature of the terahertz wave irradiated samples increased by approximately 0.5 °C instantaneously in response to the irradiation pulse, then rapidly returned to its original level. Thus, non-irradiated samples were incubated at a temperature similar to that reached during irradiation to account for the temperature increase.

**Table 1:** Parameters of terahertz wave irradiation.

	Irradiation parameters				
	Frequency (GHz)	Pulse duration (ms)	Pulse repetition (Hz)	Power (W)	Irradiation energy (mJ/s)
1. 460 GHz, 1 W	460	10	10	1	100
2. 460 GHz, 10 W	460	10	1	10	100
3. 191 GHz, 200 W	191	2.5	0.2	200	100

**Fig. 3:**
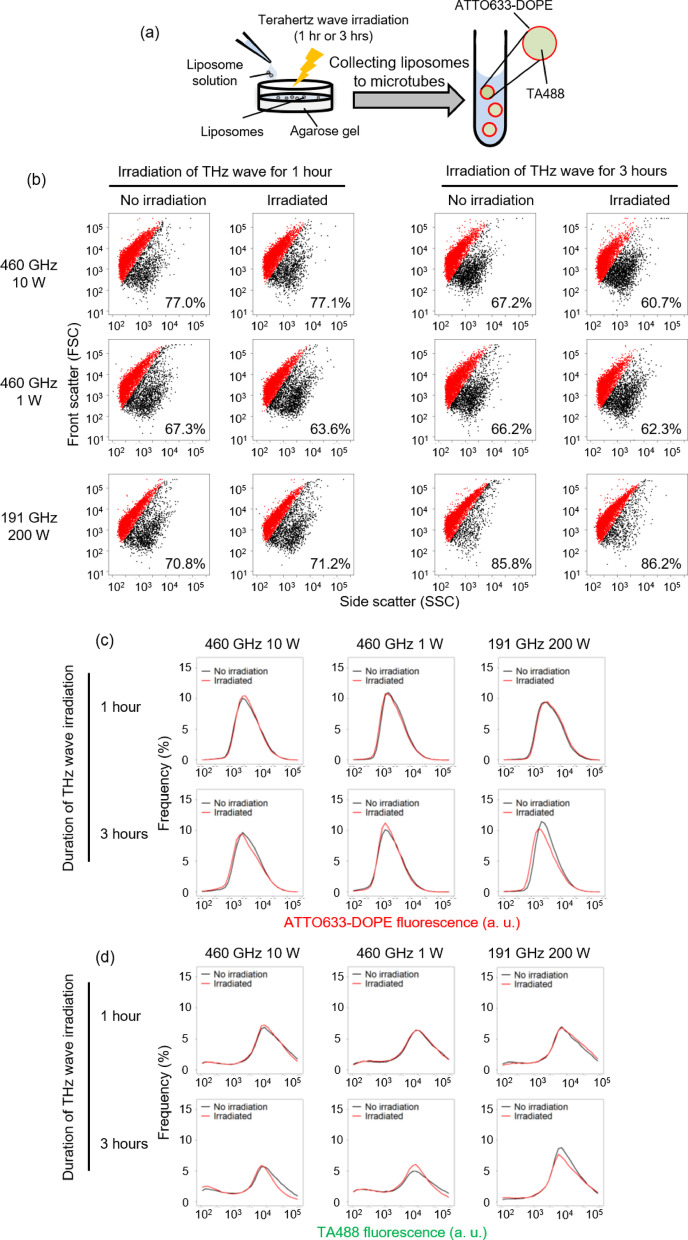
Analysis of liposome size and encapsulation materials before and after terahertz irradiation **a** Schematic representation of the experimental condition. **b** Light scattering of liposomes with or without terahertz wave irradiation were measured by FCM. Red dots represent GUVs. **c** Histogram of fluorescence of lipid marker ATTO633-DOPE lipid marker with or without terahertz irradiation. The vertical axis indicates the relative frequency. **d** Histogram of fluorescence of the TA488 internal content marker with or without terahertz irradiation. The vertical axis indicates the relative frequency.

We prepared liposomes encapsulating green fluorescent proteins, such as transferrin-Alexa 488, to assess the leakage of internal contents. To measure liposome size, we incorporated the red fluorescent Alexa 647-conjugated DOPE phospholipid (Fig. 3a). The liposomes were irradiated terahertz wave for 1 h, there were no changes in the GUV rate, liposome size, and the amount of internal fluorescent protein compared to the non-irradiated condition (Fig. 3b–d, irradiation of THz wave for 1 h). On the other hand, a slight decrease in the GUV rate, membrane fluorescence, and inner fluorescence intensity was observed under the 460 GHz 10 W condition after 3 h of irradiation (Fig. 3b, irradiation of THz wave for 3 h, 460 GHz 10 W), which was not observed in 460 GHz 1 W condition. Under the 191 GHz, 200 W condition, the percentage of GUVs remained unchanged, while the peaks of membrane fluorescence (Fig. 3c, 191 GHz 200 W, 3 h) and inner content fluorescence (Fig. 3d, 191 GHz 200 W, 3 h) shifted to the left. The membrane fluorescence intensity measured by flow cytometry is proportional to liposome size^24^, suggesting that prolonged irradiation with 191 GHz, 200 W terahertz waves led to liposome shrinkage. Additionally, the fluorescence intensity of the encapsulated fluorescent protein slightly decreased. Given that the temperature change during terahertz wave irradiation was only around 0.5–1 °C, it is likely that molecular leakage from the internal fluid occurred rather than fluorescent protein inactivation. These findings suggest that prolonged exposure to 191 GHz, 200 W terahertz waves induce structural damage to the liposome compartment.

We then observed the liposomes using fluorescence microscopy to confirm their morphology. After incubating the liposomes on an agarose gel for 1–3 h at the same temperature as the terahertz wave irradiation conditions, the images showed that the green fluorescent protein was enclosed within the red fluorescent membrane (Fig. S4, No irradiation). These images were consistent with those of liposomes prepared in a previous report, which were not incubated on an agarose gel^8^. In addition, when the liposomes were observed after being irradiated for 1–3 h under each terahertz wave irradiation condition, no differences were observed between the irradiated and non-irradiated conditions under microscopic observation (Fig. S4). According to these data, we concluded that terahertz wave irradiation does not cause damage to the membrane compartments after 1 h, and the liposomes remain stable. However, irradiation for 3 h results in a slight decrease in size without changes in liposome shape (Fig. 3c, d, Fig. S4). Since the liposomes were placed on the gel for 3 h, with or without irradiation, and the temperature was controlled, it is likely that the liposome shape was affected directly by the terahertz waves interacting with the lipid membrane or indirectly through water molecules in the inner fluid, rather than by environmental factors such as drying or temperature. The greater reduction in GUV size observed at the 191 GHz, 200 W condition compared to the 460 GHz conditions is thought to be due to the higher peak power per pulse (W). These results suggest that it may be possible to differentiate between cell damage and effects on intracellular biochemical reactions by examining parameters such as peak power, pulse interval, pulse width, and frequency, using liposomes as model cell-like compartments. However, a more detailed examination of these parameters is necessary.

### 460 GHz terahertz irradiation suppressed transcription reaction

**Fig. 4:**
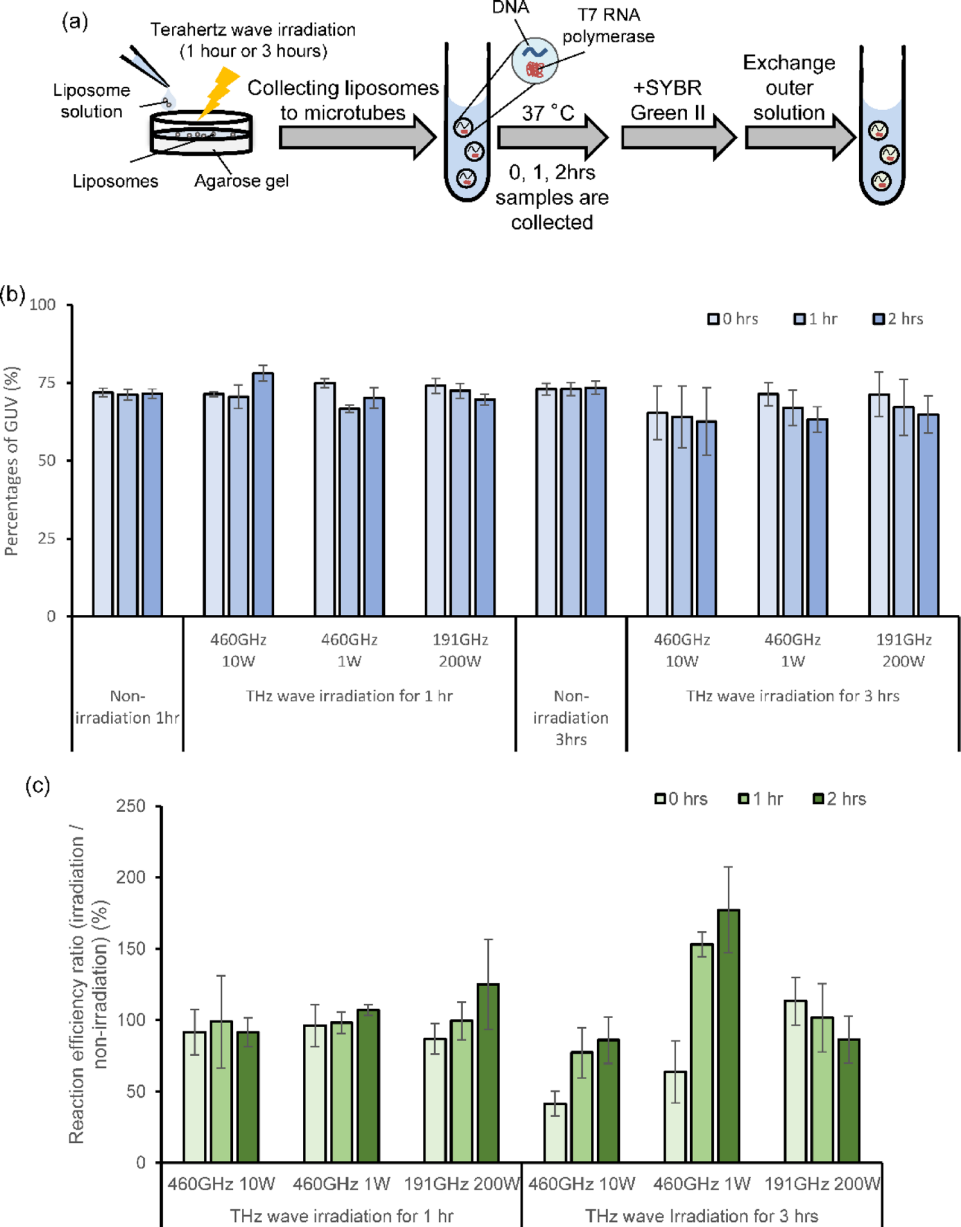
Irradiation of terahertz wave for liposomes encapsulating transcription reaction. **a** Schematic representation of the experimental condition. **b** The average ratios of GUVs collected from an agarose gel with or without terahertz wave irradiation. The error bars indicate the standard errors (*N* = 3). **c** The average of transcription efficiency ratio between irradiated liposomes and non-irradiated liposomes. The error bars indicate the standard errors (*N* = 3).

It was found that the six irradiation conditions (Table 1) did not cause significant damage to the liposomes. Next, the effects of these irradiation conditions on the transcription reaction encapsulated within liposomes were investigated. In this experiment, we prepared liposomes containing red fluorescent lipids, ATTO-633 DOPE, to measure liposome size. Terahertz wave irradiation was first applied to liposomes on gels, after which the liposomes were collected in microtubes. Then, the liposomes were incubated at 37 °C for 0, 1, and 2 h, after which SYBR Green II was added to stain the RNA, and the green fluorescence was analyzed by FCM. (Fig. 4a). The temperature of the liposomes was measured by an IR camera during terahertz wave irradiation, and the temperature of the non-irradiated liposomes was kept the same as that of the irradiated liposomes, thereby eliminating the effect of temperature. First, when irradiated for 1 h under each condition, the percentages of GUV did not change significantly immediately after irradiation (Fig. 4b, each condition in irradiation 1 h, 0 h for tube reaction) and remained stable during incubation in the tubes (Fig. 4b, irradiation 1 h, 1–2 h for tube reaction, Fig. S5a). Although the non-irradiated liposomes remained stable during incubation for 3 h on the gels, the percentages of GUV decreased during irradiation and incubation at 37 °C for the liposomes irradiated for 3 h under each condition, suggesting increased instability due to terahertz wave irradiation (Fig. 4b, each condition in irradiation 3 h, Fig S5b).

Next, we compared RNA transcription efficiency between the non-irradiated and irradiated conditions. We defined the transcription efficiency ratio (TER) as the ratio of the average fluorescence of SYBR Green II in irradiated samples to that in non-irradiated samples. A TER of 100% indicates that the average fluorescence remained unchanged with or without irradiation. First, we found that when irradiated for 1 h under each condition, there was no difference in the amount of RNA transcribed with or without irradiation (Fig. 4c hr; Fig. S6a). Under the condition of 191 GHz and 200 W, the intra-tube transfer efficiency of the irradiated sample was slightly higher than that of the non-irradiated sample, accompanied by an increase in TER. However, a Student’s t-test revealed no statistically significant difference in the average fluorescence intensity of SYBR Green II, as shown in the FCM data in Fig. S6a (Fig. 4c, 191 GHz, 200 W, 1 h irradiation, Fig. S6a). In addition, fluorescent microscopy revealed that transcriptional reactions occurred within the liposomes, with no change in RNA localization regardless of irradiation (Fig. S7a). Interestingly, we found that irradiation at 460 GHz for 3 h resulted in a decrease in RNA transcription efficiency during irradiation, regardless of the wave power (W) (Fig. 4c hrs; Fig. S6b). Notably, the subsequent increase in TER suggests that the enzyme was not inactivated and that transcription efficiency may have been higher compared to the non-irradiated samples. In particular, under the condition of 460 GHz and 10 W, the average fluorescence intensity in the FCM data (Fig. S6b) was significantly lower immediately after irradiation (*p* < 0.05, Student’s t-test). Under the condition of 460 GHz and 1 W, although the difference was not statistically significant (*p* < 0.15), a slight decrease in average fluorescence intensity was observed. These differences disappeared after 1 and 2 h of tube reaction, further suggesting that the reaction proceeded more efficiently in the irradiated samples. On the other hand, under the 191 GHz, 200 W condition, TER remained at approximately 100% immediately after irradiation. Although there was no statistically significant difference in the average fluorescence intensity of SYBR Green II between the non-irradiated and irradiated samples, the TER gradually decreased during the reaction in the tube, likely due to the leakage of liposomal contents and the reduction in liposome size, as shown in Fig. 3c and d. This resulted in a lower concentration of substrate and enzyme compared to the non-irradiated sample.。Under fluorescence microscopy, the morphology and RNA localization of these liposomes did not change significantly (Fig. S7b). These data suggest that terahertz waves non-thermally and reversibly alter the activity efficiency of transcription enzymes, with effects dependent on frequency and energy level.

## Discussions

In this study, we first established an experimental system for irradiating terahertz waves to liposomes under conditions without an outer solution (Figs. 1 and 2) and analyzed the effects of irradiation on GUVs (Fig. 3). Subsequently, we used two terahertz wave frequencies to examine the impact of various irradiation conditions (Table 1) on the efficiency of transcription reactions in liposomes (Fig. 4). GUVs are stable across a wide range of physiological temperatures. Therefore, it is notable that the membrane and inner fluorescence levels change with and without irradiation, as shown in Fig. 3. The reason for this change is not clear in this study, but since the local temperature increase due to terahertz wave irradiation is only 0.5 °C to 1 °C, and the non-irradiated samples were incubated at the same temperature as the irradiated samples, temperature is unlikely to be the cause of the change in GUV shape. Some studies have shown that bilayers composed of 1,2-dimyristoyl-sn-glycero-3-phosphocholine (DMPC) or 1,2-dimyristoyl-sn-glycero-3-phosphoryl-3-rac-glycerol (DMPG) exhibit weak absorption at 1.08 and 2.10 THz, respectively^25,26^. Additionally, a recent study indicated that irradiation with 130–150 GHz waves at 6.2 mW/cm² caused permeability changes in cationic liposome membranes composed of complex phospholipids^27^. Thus, our results suggest that prolonged irradiation may affect liposome membranes, altering their fluidity and permeability. This effect may depend more on frequency than on the peak power per pulse, but this study alone cannot determine which factors, including frequency, pulse width, or pulse spacing, are most important. Further studies are needed to clarify this.

We found that irradiation with terahertz waves at 460 GHz for 3 h reduced transcription efficiency during irradiation, as indicated by a TER value lower than 100% (Fig. 4c, 460 GHz 10 W or 1 W irradiated for 3 h, 0 h for tube reaction). However, the TER increased during the subsequent reaction in microtube (Fig. 4c, 460 GHz 10 W or 1 W irradiated for 3 h, 1 h and 2 h for tube reaction), indicating that transcription efficiency after terahertz irradiation became higher compared to the non-irradiation conditions. This effect was not observed at 191 GHz 200 W irradiation for 3 h condition (Fig. 4c). Notably, the changes in liposome size and the amount of encapsulated protein were minimal during terahertz wave irradiation of liposomes (Fig. 3). In addition, the percentages of GUV did not differ significantly with or without terahertz wave irradiation for transcription reaction (Fig. 4b, irradiation 3 h, 0 h in each condition). These results suggest that the decrease in transcription reaction efficiency during terahertz wave irradiation is not attributable to damage to the liposome compartments, but rather that terahertz waves may directly affect transcription enzymes and substrates. It has been reported that irradiation of terahertz wave affects the state of water molecules and protein hydration changed^20^. Our results suggest that during terahertz wave irradiation, the hydration state of the enzyme’s active site changes, leading to a stronger bond between the enzyme and DNA. This bond strengthening may prevent RNA polymerase from sliding and performing the elongation reaction. Consequently, the enzyme-DNA complex may accumulate, which could enhance the RNA synthesis reaction rate during the first hour when the reaction is allowed to proceed in the tubes after irradiation. The difference in transcription reactions between 1 and 3 h of irradiation is likely due to the reaction rate of T7 RNA polymerase. At 1 h, the amount of transcription product is small, making it difficult to detect changes in transcription efficiency by fluorescence (Fig. S3 28 ℃, 1 h on gel). In contrast, over 3 h, non-irradiated samples show more substantial transcriptional activity (Fig. S3, 28 ℃, 3 h on gel), allowing the suppression of transcription in irradiated samples to be more readily observed. We concluded that the 460 GHz frequency inhibits the transcription reaction, and that pulse power is also a critical factor influencing this effect. However, further investigation is required to clarify the individual contributions of parameters such as frequency, energy dose, pulse width, and pulse spacing to the modulation of biochemical reactions.

The gyrotron used in this study can change the frequency of terahertz waves discretely in the range of 160 to 460 GHz. The output power can be varied from 1 W to a maximum of 200 W, depending on the oscillation frequency. It has been reported that irradiation at 460 GHz with a 10 ms pulse and 0.6 W/cm² for 30 min inhibited cell division during irradiation^5^, and that germination elongation of soybean seeds was observed after 20 min of irradiation at 110 GHz, 10 mW^28^. Thus, while the effects of irradiation on various organisms under different conditions have been observed, there is still no unified understanding of the optimal experimental conditions. Since all cells rely on transcription and translation, analyzing the effects of terahertz wave irradiation on these processes encapsulated in GUVs could help identify the critical frequencies and parameters for experiments involving organism irradiation, as well as the specific conditions under which terahertz wave irradiation exerts its effects. Therefore, the experimental system we have developed for irradiating liposomes provides a novel approach to elucidate the effects of terahertz waves on biological organisms.

## Materials and methods

### Materials

1-Palmitoyl-2-oleoyl-*sn*-glycero-3-phosphocholine (POPC) was derived from Nippon Fine Chemical (Osaka, Japan). Liquid paraffin (0.86–0.89 g/mL at 20 °C) and chloroform were purchased from Wako (Osaka, Japan). ATTO-633 conjugated 1,2-dioleoyl-*sn*-glycero-3-phosphoethanolamine (DOPE) was purchased from ATTO-TEC (Siegen, Germany). Transferrin Alexa Fluor 488 conjugate (TA488) and Transferrin Alexa Fluor 647 conjugate (TA647) were purchased from Life Technologies (Carlsbad, CA, USA). T7 RNA polymerase was purchased from Takara Bio (Shiga, Japan).

### Liposome preparation

The water-in-oil emulsion transfer method was described in a previous report^8,21,29^. Briefly, POPC was dissolved in chloroform at a concentration of 0.1 mg/µL and mixed with liquid paraffin to create a 5 mg/mL lipid solution, which was then heated at 80 °C for 30 min. For fluorescence microscopy, ATTO-633 DOPE was added to achieve a final concentration of 5 µg/mL. Following the preparation of the lipid mixture, 20 µL of the inner solution (50 mM HEPES-KOH (pH 8.0), 350 mM potassium glutamate, 500 mM sucrose, 2 mM NTPs, 5 mM dithiothreitol (DTT), 1× T7 RNA polymerase buffer, T7 RNA polymerase, and template DNA under the indicated conditions) was added to 400 µL of the lipid mixture. The solution was mixed for 20 s using a vortex mixer, followed by tapping for an additional 20 s. This mixing process was repeated three times. The resulting water-in-oil emulsion was incubated on ice for 10 min. Subsequently, 400 µL of the emulsion was carefully layered on top of 200 µL of the outer solution (50 mM HEPES-KOH, 350 mM potassium glutamate, and 1 M glucose) in a new tube and incubated on ice for 10 min. The tube was then centrifuged at 18,000 × g for 30 min at 4 °C. The liposome pellet was collected by piercing a hole in the bottom of the tube with a needle. The collected liposomes were further centrifuged at 18,000 × g for 5 min. Finally, the liposome pellet was resuspended in incubation buffer (50 mM HEPES-KOH and 1 M glucose).

### Template DNA preparation

Template DNA for T7 RNA polymerase reaction in liposome was prepared as described previously^30^. Briefly, a DNA encoding ribosome binding site and LwCas13a was amplified from pC019 ^31^ (Addgene plasmid #91909) by PCR using primers 1 and 3 (described below) and then, the amplified fragments were used for PCR amplification of the template DNA encoding the T7 promoter, ribosome binding site, and LwCas13a using primers 2 and 3.

Primer 1: AAGGAGATATACCAATGAAAGTGACCAAGGTCGACGGCA.

Primer 2: ACATGGATCCGAAATTAATACGACTCACTATAGGGAGACCACAACGG.

TTTCCCTCTAGAAATAATTTTGTTTAACTTTAAGAAGGAGATATACCA

Primer 3: GCTAGGATCCTAGTTATTCATTATTCCAGGGCCTTGTACTCGAACATCACT.

### Terahertz-irradiation

The prepared liposome solution was gently loaded onto an agarose gel (50 mM HEPES-KOH, 1.5 M glucose, and 1% agarose) in a 3.5 mm cell culture dish (Corning, NY, USA). After drying the outer solution for 10 min at room temperature, the samples were placed on the THz irradiation stage, as shown in Fig. 1. THz waves output from the gyrotron^19^ are transmitted by a circular waveguide and irradiated as a Gaussian beam to the stage. The irradiation conditions are detailed in Table 1. At each frequency, parameters including output power, pulse duration, and pulse repetition frequency can be set arbitrarily, and are maintained stably over long-term operations.

*Measuring RNA in liposomes by SYBR.* SYBR Green II was used to stain synthesized RNA in liposomes. Before measuring fluorescence by flow cytometry (FCM) or microscopy, SYBR Green II was added to the outer solution of the liposomes to a final concentration of 1/10,000 of the original. The samples were incubated for 15 min on ice, after which the outer solution was exchanged to wash out unreacted SYBR Green II.

*Flow cytometry.* Each liposome was analyzed by flow cytometry (FCM) on a FACS Aria II (BD Biosciences, San Jose, CA, USA). Forward-scatter (FSC) and side-scatter (SSC) were obtained to collect the liposome data and the giant unilamellar liposome region was determined as previously reported^30,32^. We then measured the fluorescence of SYBR green II and TA488 which were excited with a 488 nm semiconductor laser, and the emission was detected with a 530 ± 15 nm band-pass filter (FITC-A). In addition, we measured the fluorescence of ATTO633-DOPE lipid and TA647, which was excited with a HeNe laser (633 nm laser), and emission was detected with a 660 ± 10 nm band-pass filter (APC-A).

### Fluorescence microscopy

Images of liposomes were obtained using an Axio Observer Z1 microscope (Carl Zeiss) equipped with a 63× Plan-Neofluar objective lens (NA = 1.4). The intensity of SYBR green II and TA647 was excited with an LED (470 nm) and measured with a detector set to a 525 ± 25 nm bandpass filter. ATTO633-DOPE was excited with an LED (590 nm) and measured with a detector set to a 629 ± 31 nm bandpass filter.

## Electronic supplementary material

Below is the link to the electronic supplementary material.

Supplementary Material 1

## Data Availability

The datasets used and/or analyzed during the current study are available from the corresponding author on reasonable request.
