# Supplementary material for: 0.46 Terahertz wave irradiation inhibit transcription reaction in liposomes
Source: Sci Rep. 2025 May 28;15:18729. doi: 10.1038/s41598-025-03869-w (PMC12119987; doi:10.1038/s41598-025-03869-w)
Supplement: Supplementary file 1 — Supplementary Material 1 [file 41598_2025_3869_MOESM1_ESM.docx]

**0.46 terahertz wave irradiation inhibit transcription reaction in liposomes**

**Supplemental Figures**


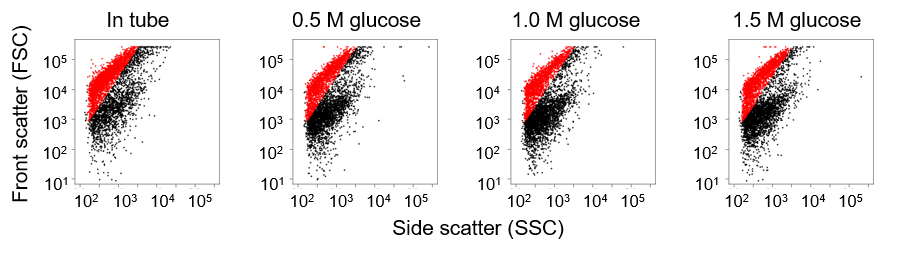


**Figure S1. FCM analysis of GUV ratio before and after applied on the agarose gel.** Light scatterings of liposomes with or without terahertz wave irradiation were measured by FCM. Red dots represent GUVs.


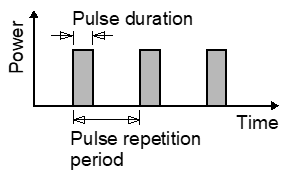


**Figure S2. Schematic illustration of irradiation parameters.** Schematic representation of parameters for irradiating terahertz wave. Indicated powers (W) of terahertz wave was irradiated as indicated pulse duration (ms) with intervals defined as pulse repetition frequency (Hz).

**Figure S3 Transcription reaction in liposomes on agarose gel.** The average of SYBR green II fluorescence of the GUVs measured by using FCM for three independent experiments were shown. The error bars indicate standard errors (N = 3).


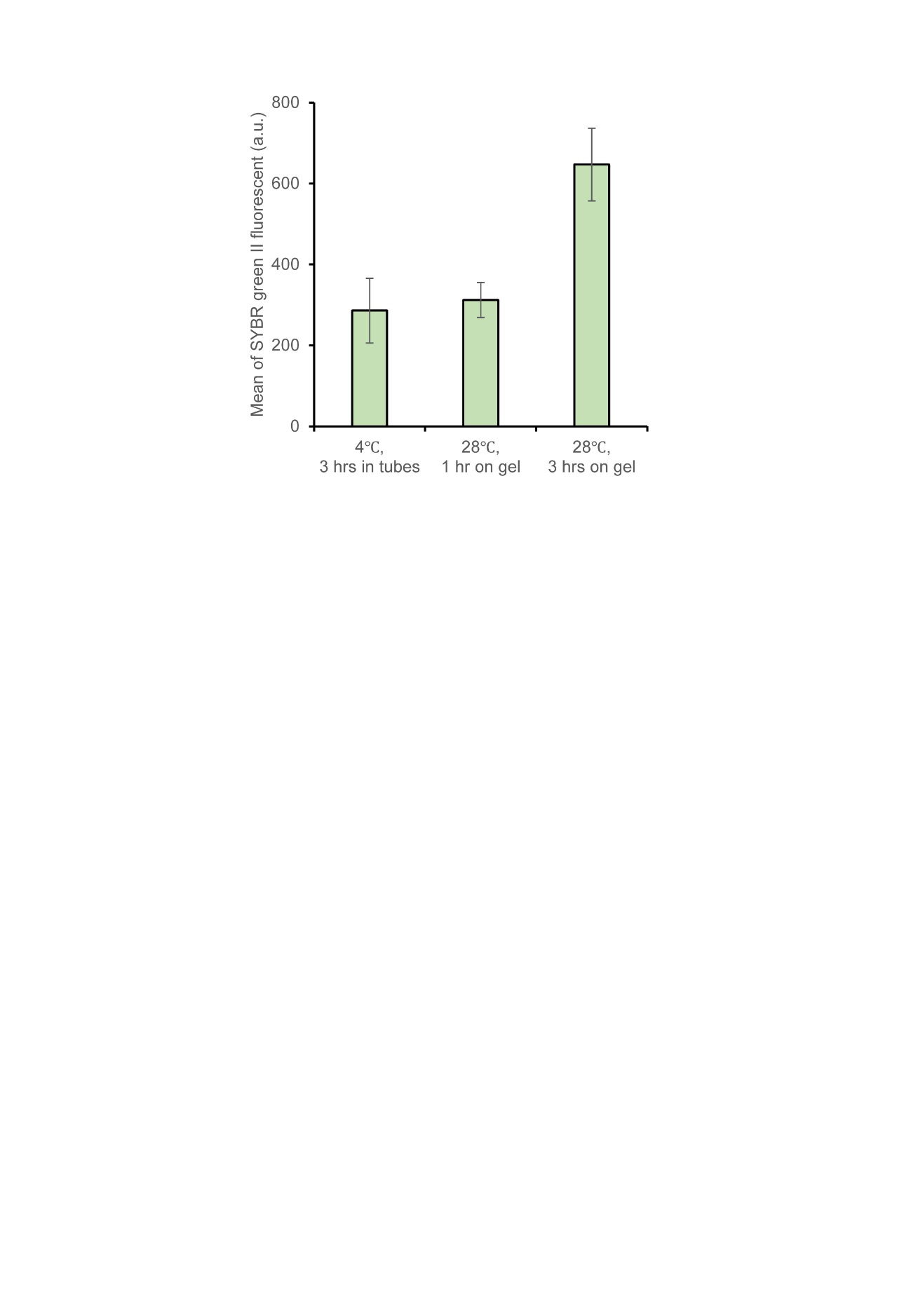


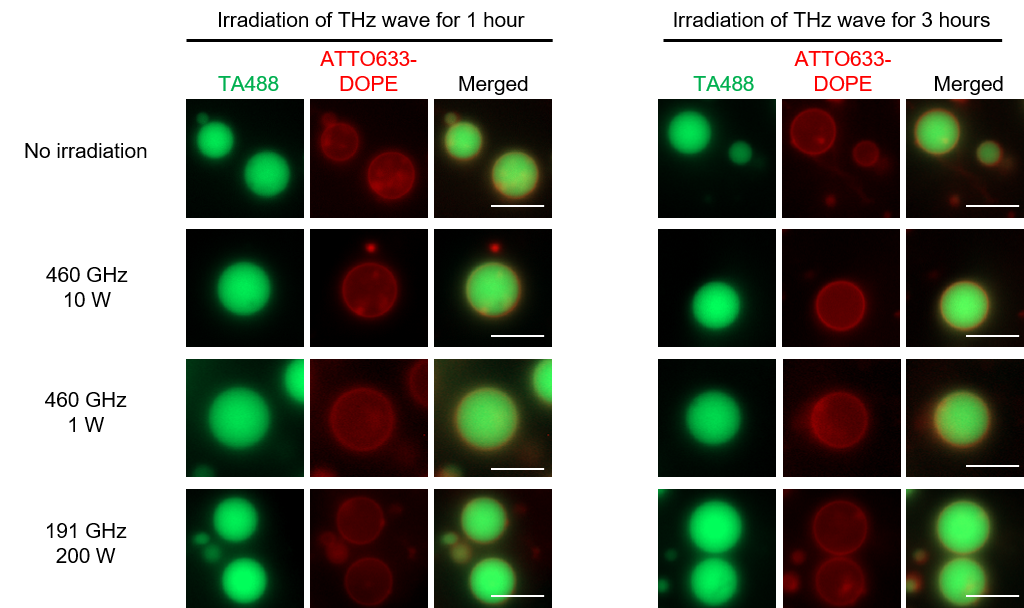


**Figure S4 The fluorescence images of liposomes with or without terahertz wave irradiation.** Fluorescence microscopy images of liposomes collected from an agarose gel. The fluorescence of TA488 (green) and ATTO633-DOPE (red) is shown. (Scale bar, 10 μm.)

**
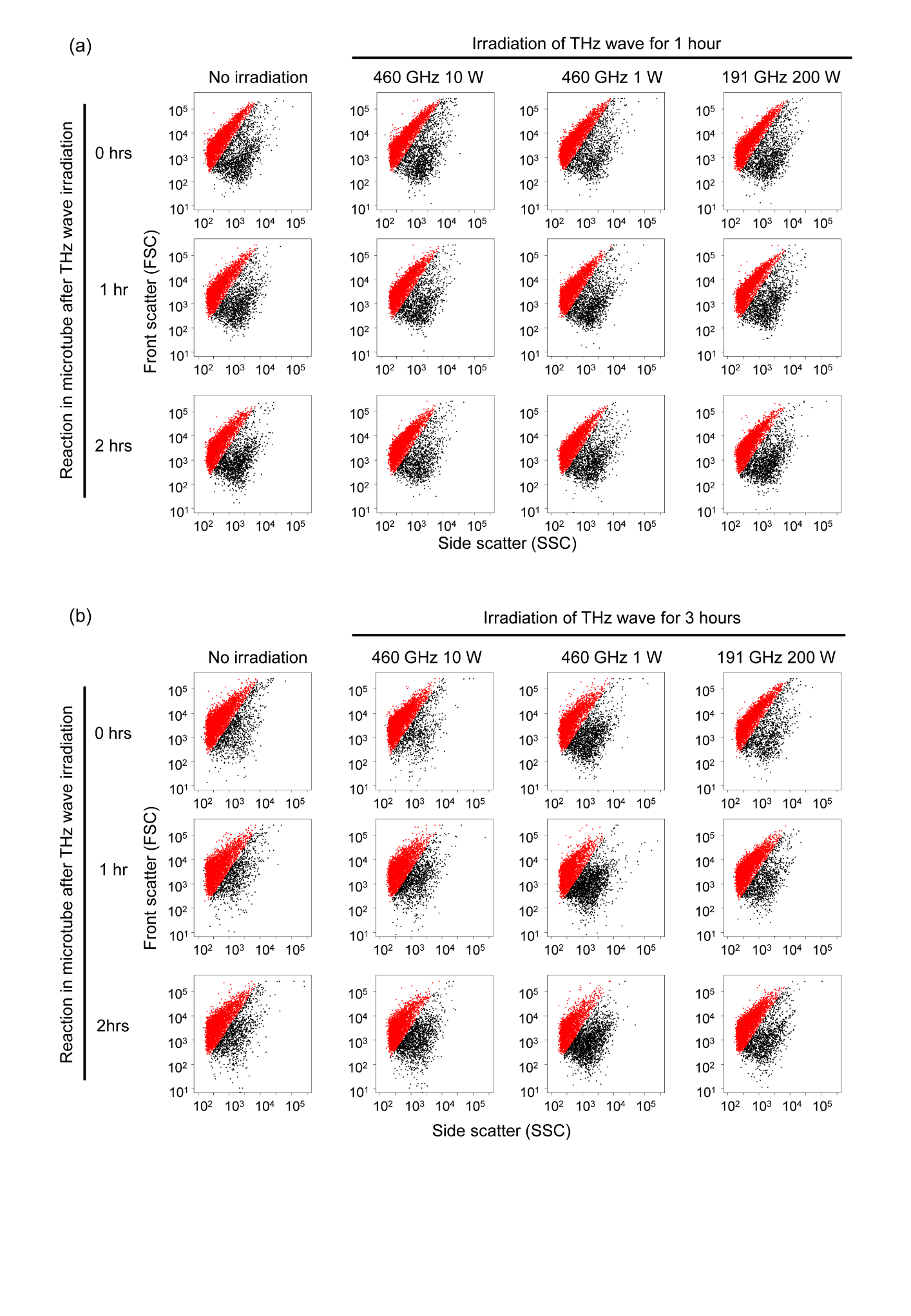
Figure S5 FCM analysis of the GUV ratio after collection from agarose gel with or without the indicated THz wave irradiation**. Light scatterings of liposomes with or without terahertz wave irradiation for 1 hour at the indicated THz wave conditions were measured by FCM. Red dots represent GUVs. (a) Liposomes are irradiated THz wave for 1 hr (b) Liposomes are irradiated THz wave for 3 hrs.


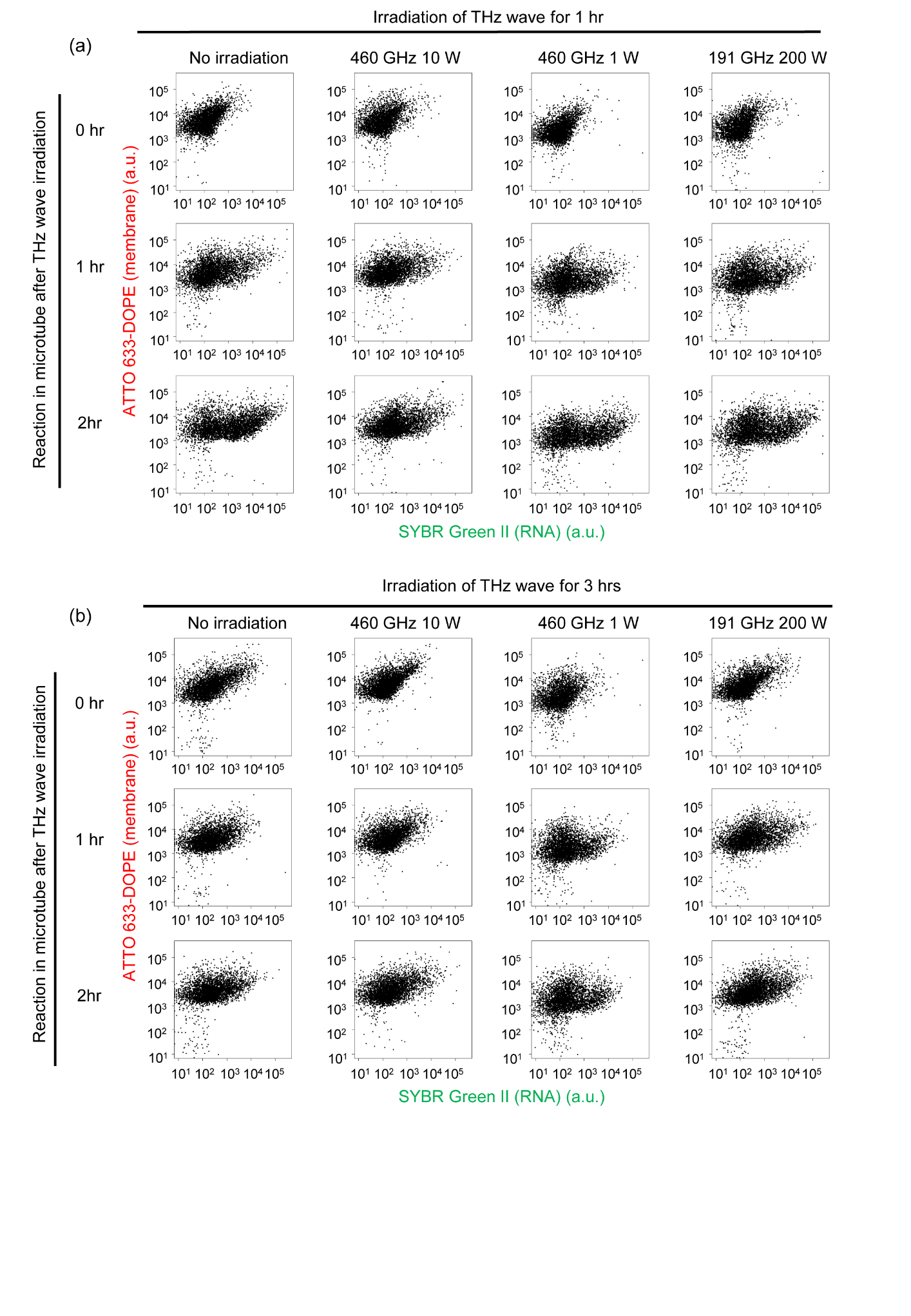
 **Figure S6. FCM analysis of fluorescence after collection from agarose gel with or without the indicated THz wave irradiation.** Fluorescence of membrane marker (ATTO633-DOPE) and RNA (SYBR Green II) in liposomes with or without terahertz wave irradiation under the indicated conditions were measured by FCM. (a) Liposomes are irradiated THz wave for 1 hr (b) Liposomes are irradiated THz wave for 3 hrs.


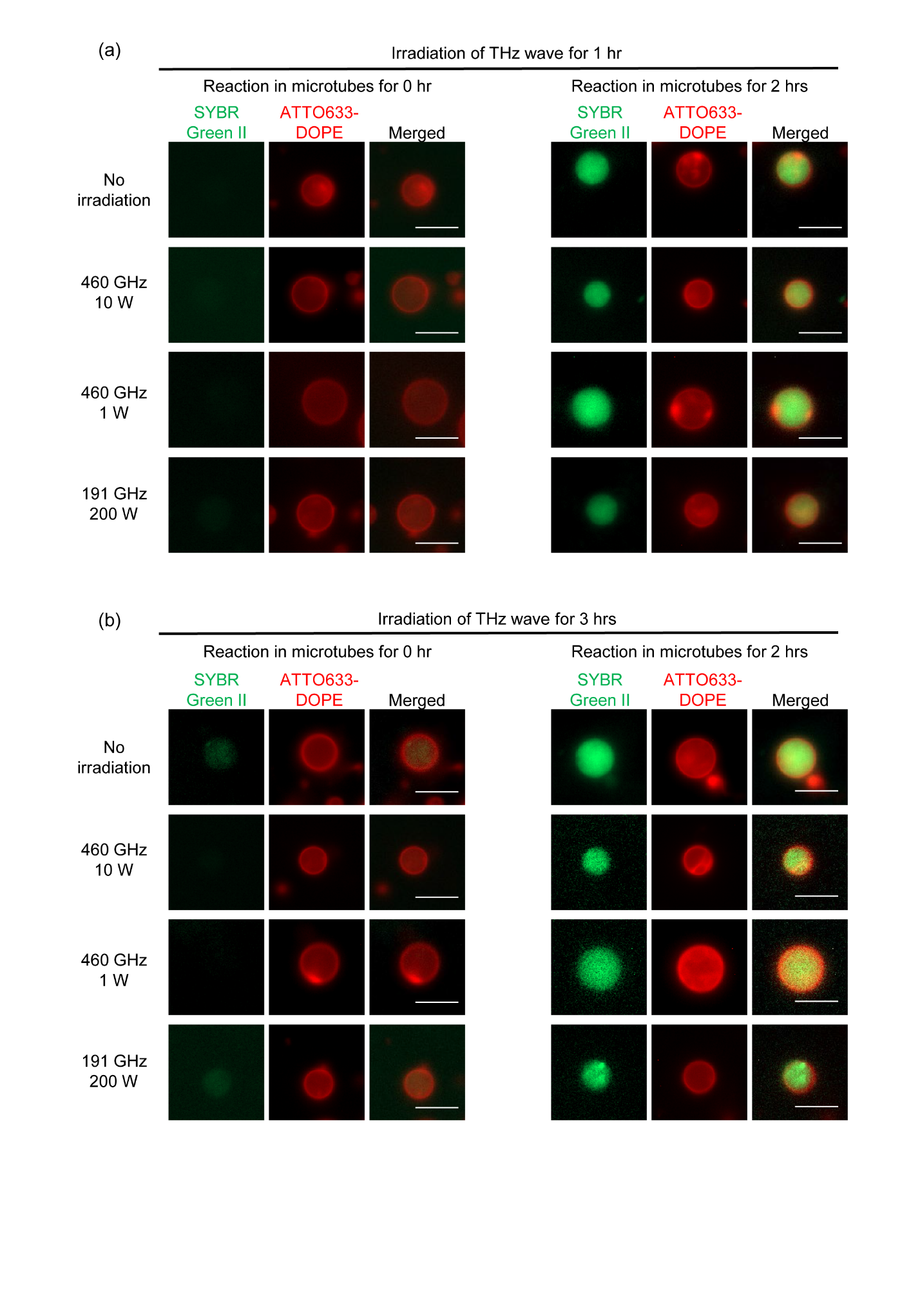
**Figure S7. The fluorescence images of liposomes with or without terahertz wave irradiation.** Fluorescence microscopy images of liposomes, collected from an agarose gel after terahertz wave irradiation under the indicated conditions. The fluorescence of TA488 (green) and ATTO633-DOPE (red) is shown. (Scale bar, 10 μm.). 0 hr and 2hr indicate reaction times after THz wave irradiation.
